# Supplementary material for: Fabrication and Thermal Performance of a Polymer-Based Flexible Oscillating Heat Pipe via 3D Printing Technology
Source: Polymers (Basel). 2023 Jan 12;15(2):414. doi: 10.3390/polym15020414 (PMC9863260; doi:10.3390/polym15020414)
Supplement: Supplementary file 1 [file polymers-15-00414-s001.zip › polymers-2061103-supplementary.pdf]

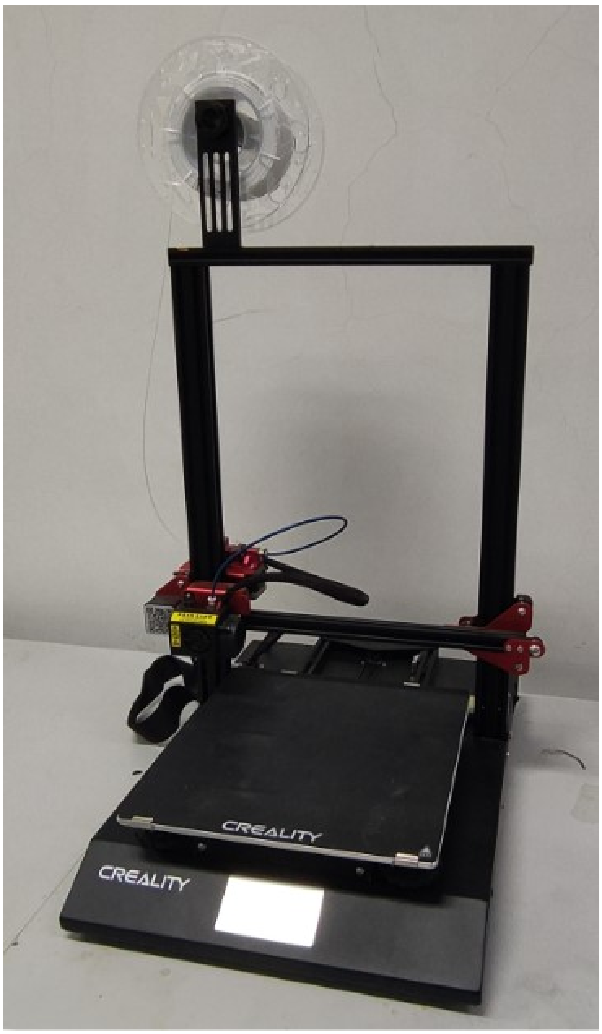

Figure S1. Optical image of the 3D printer.

Note S1: Key features of the 3D printer

The 3D printer with an accuracy of ±0.1 mm was used to fabricate the FOHP, and its build volume was 220 mm x 220 mm x 250 mm. The diameter of nozzle, maximum hot bed temperature, and maximum printing speed were 0.4 mm, 110 ^{o}C, and 200 mm/s, respectively. The layer thickness was 0.1~0.4mm.
